# Strengthening Undergraduate Medical Education for Inclusive Health Care for People With Down Syndrome and Intellectual and Developmental Disabilities in Medical Schools: Protocol for a Scoping Review

**DOI:** 10.2196/80280

**Published:** 2026-03-16

**Authors:** Isabela Amaral de Almeida Bistafa, Marco Antonio Ribeiro Filho, Ana Caroline dos Santos Costa, Ana Flávia Pimenta Saad, Rauer Ferreira Franco, Janaína Aparecida de Sales Floriano, Emerson Roberto dos Santos, João Daniel de Souza Menezes, Matheus Querino da Silva, Daniele Nunes Longhi Aleixo, Eliana Fazuoli Chubaci, Andressa Karina Stefani, William Donegá Martinez, Pedro Belchior da Silveira Junior, Ana Mirtes Zecchin, Nathalia Bavaresco Gonçalves Cristóvão, Alex Bertolazzo Quitério, Renato Mendonça Ribeiro, Denise Cristina Móz Vaz Oliani, Antônio Hélio Oliani, Maysa Alahmar Bianchin, Luís Cesar Fava Spessoto, Rita de Cássia Helú Mendonça Ribeiro, Júlio César André

**Affiliations:** 1 Center for Studies and Development of Health Education – CEDES, Avenida Brigadeiro Faria Lima, São Jose do Rio Preto, São Paulo, 5416, Brazil, 55 996140498; 2 Ribeirão Preto School of Nursing, Av. Dr. Enéas de Carvalho Aguiar, 419, Brazil; 3 University Hospital Center Cova da Beira, University of Beira Interior, Covilhã, Portugal; 4 Faculdade de Medicina de São José do Rio Preto, Sao Jose do Rio Preto, Brazil

**Keywords:** medical education, Down syndrome, intellectual disability, developmental disability, scoping review protocol

## Abstract

**Background:**

Health inequities represent a persistent and multifaceted challenge, particularly pronounced for individuals with intellectual and developmental disabilities (IDDs), including Down syndrome (DS). This population frequently faces systemic barriers to care and is at higher risk of adverse health outcomes*.* Despite advances, gaps persist in health care professionals’ training for caring for this population. Undergraduate medical education constitutes a crucial component in addressing these disparities and promoting inclusive care, especially through practical experiences for future physicians.

**Objective:**

This study aims to map and synthesize evidence regarding undergraduate medical education for the care of individuals with DS and other IDDs in medical schools, identifying gaps and opportunities for enhancement in curricula and educational programs, including pedagogical strategies and experiential learning opportunities.

**Methods:**

A scoping review following the methodological frameworks by Arksey and O’Malley, Levac et al, and the Joanna Briggs Institute will be conducted. The search will be performed in electronic databases and gray literature sources using descriptors in English, Portuguese, and Spanish. Study selection will involve undergraduate medical students as the target population. Two independent reviewers will perform study selection following predefined inclusion and exclusion criteria. Data will be extracted using a standardized form and synthesized narratively, with qualitative (thematic) and descriptive quantitative analyses where appropriate. This protocol has been registered with the Open Science Framework and will adhere to the PRISMA-ScR (Preferred Reporting Items for Systematic Reviews and Meta-Analyses extension for Scoping Reviews) guidelines.

**Results:**

A comprehensive overview of current undergraduate medical education regarding the care of individuals with DS and other IDDs will be provided, informing the development of more effective and inclusive interventions and yielding insights into existing pedagogical strategies, practical learning opportunities, and medical students’ knowledge and attitudes toward this population, providing a foundation for evidence-based recommendations.

**Conclusions:**

This review will address an important gap in the literature, providing insights for the improvement of undergraduate medical education and clinical practice in caring for individuals with DS and other IDDs, contributing to the development of more capable and empathetic professionals. By systematically mapping the current landscape and identifying specific needs, this protocol lays the groundwork for transformative changes in medical curricula worldwide, ultimately enhancing health outcomes for this vulnerable population.

## Introduction

Health inequities represent a persistent and multifaceted challenge, with studies indicating that they affect a significant portion of patients [1] and are particularly pronounced for individuals with intellectual and developmental disabilities (IDDs), including Down syndrome (DS) [2,3]*.* This population frequently faces systemic barriers to care and is at higher risk of adverse health outcomes, which is exacerbated by the need for more intensive interactions with the health care system [4]. Despite advances in inclusion policies and disability rights, a significant gap persists in research on health care professionals’ self-efficacy in caring for patients with IDDs [5]. This lack of professional preparation can perpetuate inequalities in access and quality of care, contributing to inadequate health outcomes [6]. Furthermore, people with IDDs face systemic barriers such as limited access to preventive health services and unfavorable social determinants [7]. Including the perspective of people with disabilities is essential to understand their unique experiences and challenges; however, a medicalized view still predominates, obscuring the structural and social barriers faced by this population [2]. A critical approach to disability in medical education can help contextualize these experiences and promote transformation in health care systems toward equity and inclusion [8]. The inequities faced by people with IDDs in health care are multifaceted and can be exacerbated by factors such as lack of professional confidence and skills, limited resources, and explicit and implicit biases [9]. These biases may include misconceptions about the quality of life and dignity of people with disabilities, leading to disparities in care [10].

Medical education holds a fundamental responsibility in shaping the perspectives, knowledge, and skills of future physicians to effectively address these disparities. It is within medical schools that basic science, essential clinical skills, and ethical postures toward diverse patient populations are established and developed. However, theoretical knowledge or superficial exposure to specific topics alone is insufficient. The ability to provide truly person-centered and equitable care, particularly for populations that demand individualized and multifaceted approaches, such as those with IDDs, heavily depends on specific training adapted to their unique needs.

Beyond the formal curriculum, training physicians through experiential learning in caring for people with DS and other IDDs is of profound importance and recognized as fundamental [11,12]*.* Direct exposure and interaction—a crucial “lived experience”—with individuals with these conditions throughout the medical school curriculum is fundamental. This practical experience offers students the following opportunities: developing empathy and understanding by directly interacting with people with IDDs and their families to transcend purely clinical descriptions and understand the daily realities and challenges and, above all, the capabilities and potential of each individual; challenging preconceived notions and biases through direct contact to help deconstruct stereotypes and misconceptions about quality of life, autonomy, and dignity, promoting a more humane and less pathologizing view; building confidence and practical skills via supervised practice in relevant environments to allow students to feel more confident in communicating, conducting adapted clinical assessments, and effectively planning care for this population; and understanding the integral context by experiencing services or community interactions that can reveal the complex interaction among health conditions, social determinants, family and community support, and environmental barriers that impact the health and well-being of people with IDDs.

Therefore, investigating the current state of medical education, and especially the inclusion of opportunities for meaningful practical experience, is of vital importance to identify how well future physicians are being prepared to care for people with IDDs in an inclusive and competent manner. This research is not merely descriptive but a fundamental step toward supporting the need for curricular and pedagogical changes.

Despite growing awareness of these issues, formal education on caring for people with IDDs remains limited in health curricula. A previous systematic review [8] highlighted the scarcity of research in this area, primarily focusing on postgraduate training and the urgent need for interprofessional and competency-based educational programs. Worldwide, various initiatives and frameworks have emerged to address this gap. These programs should adopt a critical perspective on disability and an equity and inclusion framework to guide curriculum development [13]. Innovative educational interventions such as immersive curricula and interprofessional initiatives have shown promising results in reducing prejudice and improving knowledge and skills among health care professionals [3,14]. These approaches can promote transformative learning and improve the quality of care provided to people with IDDs [11]. However, the effective implementation of these interventions requires institutional commitment and cultural change in health schools. Barriers such as lack of time, limited resources, and resistance to change can hinder the integration of these contents into already overburdened curricula [15]. Furthermore, assessing the impact of these initiatives on clinical practice and long-term health outcomes remains a challenge [12]. Despite these challenges, there are inspiring examples of successful educational programs. In the United States, a national consensus on disability health competencies was developed through extensive collaboration [16], establishing a model for training more capable and empathetic health care professionals. Similar initiatives are crucial at an international level to ensure a comprehensive and standardized approach aligned with global best practices*.*

In undergraduate medical education, there is a recognized scarcity of studies specifically focused on training future physicians to care for people with IDDs, including those with DS. This knowledge and research gap makes it difficult to precisely identify specific challenges and concrete opportunities for improvement in medical school curricula. Therefore, this scoping review differentiates itself from existing systematic reviews by focusing exclusively on undergraduate training and aims to map and synthesize the available evidence on medical education in this area, providing essential insights for the urgent enhancement of training and clinical practice in a global context. A preliminary search conducted in key databases (eg, PubMed and Web of Science) confirmed the absence of existing scoping or systematic reviews that comprehensively address medical education specifically for the care of individuals with DS and other IDDs within the undergraduate medical school context, thus affirming the unique contribution and pressing need for this study.

The specific objectives of this review include (1) identifying the presence of content related to the care of people with DS and other IDDs in undergraduate medical school curricula; (2) assessing the knowledge and attitudes of medical students toward this population; (3) identifying gaps and opportunities for improvement in medical training, including the identification of pedagogical strategies and opportunities for practical experience; and (4) providing evidence-based recommendations for the development and implementation of more comprehensive, effective, and inclusive educational programs.

The results of this review are expected to contribute significantly to the advancement of medical education and clinical practice in caring for people with IDDs in undergraduate medical training, promoting a more inclusive, equitable, compassionate, and evidence-based approach. Additionally, this review has the potential to identify knowledge gaps and guide future research in this area, strengthening the evidence base for policy, curricular, and pedagogical decisions.

## Methods

### Overview

This paper constitutes the protocol for a scoping review. A review protocol is a pre-established plan that details the methodology to be followed in conducting a systematic or scoping review. Its importance lies in ensuring transparency, reducing the risk of bias, guiding the research team, and allowing for critical evaluation of the method even before data collection begins. This scoping review will follow the methodological frameworks proposed by Arksey and O’Malley [17], Levac et al [18], and the Joanna Briggs Institute (JBI) [19], as described in the following sections.

### Protocol Registration

This protocol has been registered with the Open Science Framework (OSF Z5MDB). It will adhere to the PRISMA-ScR (Preferred Reporting Items for Systematic Reviews and Meta-Analyses extension for Scoping Reviews) guidelines to ensure transparency and high-quality reporting [20,21Checklist 1]***.***

### Eligibility Criteria (Inclusion and Exclusion)

Studies will be included in this scoping review based on the population, concept, and context framework.

The population will be undergraduate medical students who participate in educational programs or whose knowledge and attitudes regarding the care of individuals with IDDs are assessed. This specific focus on undergraduate medical students aims to capture foundational training experiences and curricular interventions during the initial stages of medical professional development, directly addressing the core objective of this review.

The concept includes educational programs; curricula; pedagogical interventions; training initiatives; or assessments of knowledge, skills, and attitudes related to health care for people with DS and/or other IDDs. The specific mention of DS is justified due to its prevalence as a genetic cause of intellectual disability often associated with distinct health profiles and societal perceptions, making it a critical exemplar within the broader IDD category. The consistent use of “IDDs” throughout the manuscript aims to harmonize terminology and encompass the wide range of conditions that fall under this umbrella, including autism spectrum disorder, neurodevelopmental disorders, and learning disabilities, ensuring a comprehensive review beyond solely intellectual impairment.

The context is medical schools or medical training environments at the undergraduate level.

Types of evidence will include original research studies (quantitative, qualitative, or mixed methods), reviews (eg, narrative, integrative, or systematic provided that they include primary data relevant to the population, concept, and context framework), experience reports, theses, dissertations, and other relevant gray literature (eg, government reports and conference proceedings). When reviews are included, data extraction will primarily focus on identifying and analyzing the primary studies cited within them. If a review itself provides primary data (eg, a descriptive analysis of curricula across institutions), it will be treated as a primary source for that specific data point, with careful consideration to avoid double counting of evidence.

No restriction will be applied to the year of publication. Studies published in English, Portuguese, and Spanish will be included. This language scope was chosen to balance comprehensive coverage with practical feasibility for translation and analysis by the review team. These languages represent a significant portion of the global scientific output relevant to medical education and disability, allowing for a broad international perspective while managing resources effectively.

### Search Strategy

#### Overview

Searches will be conducted in electronic databases and gray literature sources [22] (Table 1). Descriptors in English, Portuguese, and Spanish will be used combined with Boolean operators. The search strategy will be adapted for each database, as described in Table 1. The search will cover literature published from the inception of each database to the final search date, which is planned for December 31, 2025. Updates to the search will be performed if the review extends beyond this time frame to ensure that the most current evidence is captured.

The search process will follow the 3-stage strategy recommended by the JBI for scoping reviews [23-28].

**Table 1.** Examples of search strategies adapted for each database.

Database	Search strategy
PubMed	(“Medical Education” [MeSH^a^] OR “Medical Training” OR “Medical Teaching” OR “Medical Curriculum” OR “Undergraduate Medical Education” OR “Medical Student Education” OR “Clinical Education” OR “Experiential Learning” OR “Curriculum Development” OR “Competency Based Education” OR “Disability Education” OR “Inclusive Health” OR “Communication Training”) AND (“Down Syndrome” [MeSH] OR “Trisomy 21” OR “Intellectual Disability” [MeSH] OR “Cognitive Impairment” OR “Developmental Disability” OR “Learning Disability” OR “Autism Spectrum Disorder” OR “Neurodevelopmental Disorder” OR “Mental Retardation”)
Scopus	TITLE-ABS-KEY((“Medical Education” OR “Medical Training” OR “Medical Teaching” OR “Medical Curriculum” OR “Undergraduate Medical Education” OR “Medical Student Education” OR “Clinical Education” OR “Experiential Learning” OR “Curriculum Development” OR “Competency Based Education” OR “Disability Education” OR “Inclusive Health” OR “Communication Training”) AND (“Down Syndrome” OR “Trisomy 21” OR “Intellectual Disability” OR “Cognitive Impairment” OR “Developmental Disability” OR “Learning Disability” OR “Autism Spectrum Disorder” OR “Neurodevelopmental Disorder” OR “Mental Retardation”))
Embase	('medical education’/exp OR ’medical training’ OR ’medical teaching’ OR ’medical curriculum’ OR ’undergraduate medical education’ OR ’medical student education’ OR ’clinical education’ OR ’experiential learning’ OR ’curriculum development’ OR ’competency based education’ OR ’disability education’ OR ’inclusive health’ OR ’communication training’) AND ('Down syndrome’/exp OR ’Trisomy 21' OR ’intellectual disability’/exp OR ’cognitive impairment’ OR ’developmental disability’ OR ’learning disability’ OR ’autism spectrum disorder’ OR ’neurodevelopmental disorder’ OR ’mental retardation’)
Cochrane Library	(((“Medical Education” OR “Medical Training” OR “Medical Teaching” OR “Medical Curriculum” OR “Undergraduate Medical Education” OR “Medical Student Education” OR “Clinical Education” OR “Experiential Learning” OR “Curriculum Development” OR “Competency Based Education” OR “Disability Education” OR “Inclusive Health” OR “Communication Training”)) AND ((“Down Syndrome” OR “Trisomy 21” OR “Intellectual Disability” OR “Cognitive Impairment” OR “Developmental Disability” OR “Learning Disability” OR “Autism Spectrum Disorder” OR “Neurodevelopmental Disorder” OR “Mental Retardation”))):ti,ab,kw
Biblioteca Virtual de Saúde	(“Educação Médica” OR “Graduação Médica” OR “Ensino Médico” OR “Currículo Médico” OR “Formação Médica de Graduação” OR “Educação de Estudantes de Medicina” OR “Educação Clínica” OR “Aprendizagem Experiencial” OR “Desenvolvimento Curricular” OR “Educação Baseada em Competências” OR “Educação em Deficiência” OR “Saúde Inclusiva” OR “Treinamento em Comunicação”) AND (“Síndrome de Down” OR “Trissomia 21” OR “Deficiência Intelectual” OR “Deficiência Cognitiva” OR “Deficiência do Desenvolvimento” OR “Dificuldade de Aprendizagem” OR “Transtorno do Espectro Autista” OR “Transtorno do Neurodesenvolvimento” OR “Retardo Mental”)
Web of Science	TS=(“Medical Education” OR “Medical Training” OR “Medical Teaching” OR “Medical Curriculum” OR “Undergraduate Medical Education” OR “Medical Student Education” OR “Clinical Education” OR “Experiential Learning” OR “Curriculum Development” OR “Competency Based Education” OR “Disability Education” OR “Inclusive Health” OR “Communication Training”) AND TS=(“Down Syndrome” OR “Trisomy 21” OR “Intellectual Disability” OR “Cognitive Impairment” OR “Developmental Disability” OR “Learning Disability” OR “Autism Spectrum Disorder” OR “Neurodevelopmental Disorder” OR “Mental Retardation”)

a MeSH: Medical Subject Headings.

#### Step 1: Limited Initial Search

A preliminary search will be conducted in 2 relevant databases (eg, PubMed and Web of Science) to identify keywords and index terms (eg, MeSH [Medical Subject Headings] terms and Emtree terms) that best describe the phenomenon of interest. This initial search will help refine the comprehensive search strategy.

#### Step 2: Comprehensive Search

On the basis of the keywords and index terms identified in step 1, a comprehensive search strategy will be developed and executed in all selected databases listed in Table 1 (Embase, PubMed, Cochrane Library, Biblioteca Virtual de Saúde, Scopus, and Web of Science), as well as in gray literature sources. The strategy will use a comprehensive network of synonyms and related terms, including controlled vocabulary (eg, MeSH terms in PubMed and Emtree terms in Embase) and free-text terms. Adjacency and proximity operators (eg, NEAR/x and ADJ/x) will be used where appropriate to increase precision. The search terms will focus on core concepts of medical education and the target population, incorporating terms such as “disability education,” “competency,” “inclusive health,” and “communication training” within the educational component. For the population component, terms will be expanded to include various forms of developmental disabilities and historical terminology for maximal retrieval, such as “autism spectrum disorder,” “neurodevelopmental disorder,” “learning difficulties,” and “mental retardation” (used historically), to ensure a broad capture of relevant studies and avoid a narrow focus solely on cognitive decline related to aging. An example of the search strategy adapted for each database is provided in Table 1.

#### Step 3: Reference List Screening

The reference lists of all studies finally included in the review will be manually screened to identify any additional relevant articles not captured by the electronic searches.

### Information Sources

Searches will be conducted in electronic databases and gray literature sources, including institutional repositories (eg, OpenGrey and ProQuest Dissertations and Theses Global), websites of relevant organizations (eg, World Health Organization and American Association on Intellectual and Developmental Disabilities), governmental reports (eg, national health ministries’ guidelines), accreditation body curricula (eg, Association of American Medical Colleges and World Federation for Medical Education), and competency frameworks from professional associations.

### Study Selection and Data Extraction

The selection process will be conducted independently by 2 reviewers. Initial deduplication of search results will be performed using automated tools (eg, EndNote and Rayyan) before screening, followed by a manual check for residual duplicates. The process will follow 2 phases:

- Title and abstract screening—all retrieved records will be screened by title and abstract against the predefined inclusion and exclusion criteria using the Covidence software (Veritas Health Innovation). Potentially relevant studies will be moved to the next phase.
- Full-text review—the full texts of studies considered potentially relevant will be retrieved and assessed against the inclusion and exclusion criteria. Any disagreements between the 2 independent reviewers at any screening stage will be resolved through discussion and consensus. If consensus cannot be reached, a third reviewer will be consulted for final arbitration. The study selection process will be presented in a PRISMA (Preferred Reporting Items for Systematic Reviews and Meta-Analyses) flow diagram.

Data will be extracted by 2 independent reviewers using a standardized form, including information about authorship, publication year, country, objective, design, population, intervention, outcomes, and results (Multimedia Appendix 1). Data extraction will be managed using a customized spreadsheet (eg, Microsoft Excel or Google Sheets). To ensure consistency and reliability of data extraction, the standardized form will be tested on a sample of approximately 5 to 10 included studies by both reviewers before the complete extraction process. Any discrepancies during data extraction will be resolved through discussion and consensus between the 2 reviewers; if necessary, a third reviewer will be consulted to mediate.

### Data Synthesis

The data synthesis will be primarily narrative, providing a descriptive summary of the characteristics of the included studies. This will involve grouping studies by key themes, such as educational content, teaching methodologies, and evaluated outcomes. Descriptive quantitative analyses (eg, frequencies and percentages) will be performed to present general trends and distributions of study characteristics (eg, publication year, geographical location, study design, and target population). A thematic analysis approach will be used to identify, analyze, and report patterns and key messages related to educational programs, current gaps, and opportunities for improvement in medical training on the care of people with DS and other IDDs. The insights obtained from the included studies will be presented to map the existing evidence base. No meta-analysis will be conducted as it is outside the scope of a scoping review focused on mapping and synthesizing existing literature.

### Expert Consultation

Experts in the field will be consulted to validate the findings and provide additional insights. After the initial data synthesis, a panel of 3 to 5 experts in medical education and/or the care of individuals with DS and other IDDs in medical schools will be consulted. This panel will explicitly include “experts by experience,” meaning individuals with IDDs themselves and their caregivers, recognizing their invaluable insights into lived experiences and care needs. These experts will be selected based on their clinical experience, academic contributions, and advocacy work in the area. The consultation will be conducted through semistructured interviews or an online survey to (1) validate the preliminary findings and their interpretation, (2) identify additional gaps or emerging themes not captured by the literature search, and (3) gather insights on the applicability and implications of the results for medical education and clinical practice. The experts’ contribution will be integrated into the discussion section and used to inform recommendations.

## Results

The results of this review will provide a comprehensive overview of current medical training on the care of people with DS and other IDDs, identifying gaps and opportunities for improvement in curricula and educational programs. These findings can inform the development of more effective educational interventions and policies, contributing to the training of more capable and empathetic professionals in the care of this population.

An updated PRISMA-ScR flowchart will be meticulously developed and included in this section to visually and clearly represent the entire study selection process from the initial search results to the final included studies. This graphical representation will enhance the transparency and replicability of our methods, allowing readers to trace the progression of each article through the screening and eligibility phases.

## Discussion

### Anticipated Findings

The results of this review have the potential to impact medical education and clinical practice by identifying gaps and opportunities for improvement in training on the care of people with DS and other IDDs. However, some limitations can be anticipated, such as the heterogeneity of studies and the scarcity of research in the medical school context. Strategies to mitigate these limitations include adopting robust synthesis methods and consulting with experts to contextualize the findings.

In this section, a detailed and comprehensive discussion of the anticipated findings and their implications will be presented, aligning with the standards for rigorous academic protocols.

On the basis of the proposed methodology, we anticipate that this scoping review will reveal a significant gap in undergraduate medical education specifically tailored to the care of individuals with DS and other IDDs. We expect to identify that, while there is growing awareness of the importance of inclusive care, the implementation of curricular content and practical experiences remains inconsistent and insufficient across many medical institutions. Our findings are predicted to indicate a deficit in medical students’ knowledge and confidence regarding the management of health complexities and communication nuances pertinent to this population, as well as the persistence of biases or preconceived notions potentially exacerbated by the absence of meaningful learning experiences. Furthermore, we expect to delineate various pedagogical strategies and experiential learning opportunities that, although extant, may not be widely integrated or systematically evaluated.

### Comparison to Prior Work

As highlighted in the Introduction section, previous systematic reviews in this area have predominantly focused on postgraduate training or broader conceptualizations without an in-depth examination of the undergraduate medical curriculum. For instance, Adirim et al [8] conducted a systematic review focused on postgraduate medical training in IDDs. Our scoping review distinguishes itself by its exclusive focus on undergraduate medical education, thereby addressing a critical gap in the literature concerning the foundational training of future physicians. By identifying current educational content, pedagogical approaches, and student perceptions at this formative stage, we can provide a more robust basis for early and effective curricular interventions. Our findings will facilitate a direct comparison with the needs identified at more advanced training levels, pinpointing where fundamental competencies for compassionate and effective care require reinforcement.

### Strengths and Limitations of the Protocol

This protocol has several methodological strengths, including adherence to established frameworks (Arksey and O’Malley [17], Levac et al [18], and the JBI) and a comprehensive search strategy across multiple databases and gray literature sources in English, Portuguese, and Spanish. Registration with the OSF and adherence to the PRISMA-ScR guidelines promote transparency and minimize bias. The inclusion of “experts by experience” (individuals with IDDs and their caregivers) in the expert consultation panel represents a strength, ensuring that lived experiences inform the results. Limitations include the potential heterogeneity and scarcity of research focused on undergraduate medical education. This scarcity may constrain quantitative synthesis, increasing reliance on narrative analysis. The search’s confinement to selected languages may exclude relevant research. The nature of a scoping review implies that no meta-analysis will be conducted. Dependence on secondary data dictates that the quality of the extracted information is contingent upon its reporting in primary studies.

### Future Directions and Implications for Policy and Practice

Insights generated by this review may inform future research and practical initiatives. Future directions include (1) rigorous development and evaluation of innovative educational interventions (eg, immersive curricula and interprofessional programs), (2) investigation of barriers to and facilitators of curricular changes in medical schools, and (3) longitudinal studies assessing the impact of enhanced training on clinical practice and health outcomes for individuals with DS and other IDDs. The results may inform educational policies and guidelines for accreditation bodies aiming to integrate essential competencies for the care of this population into undergraduate curricula. The ultimate goal is to foster an educational ecosystem that produces more prepared, empathetic, and inclusive professionals.

### Dissemination Plan

A robust dissemination plan will be implemented. Results will be submitted for publication in leading peer-reviewed journals specializing in medical education and disability health. The findings will be presented at national and international scientific conferences and workshops. Executive summaries and communication materials will be developed for curriculum committees, professional councils, and disability advocacy organizations. Making the protocol and data available on the OSF will ensure public access and transparency. Active engagement with communities of people with disabilities and their caregivers in dissemination is intended to ensure that the research is informed by the needs derived from their lived experiences.

### Conclusions

This scoping review will address an important gap in the literature by analyzing medical training on the care of people with DS and other IDDs in medical school. The results may support improvements in curricula and educational programs, contributing to the training of more capable and empathetic professionals in the care of this population at higher risk of adverse health outcomes.

The contribution of this work is fundamental to reshaping undergraduate medical education, fostering a more inclusive and equitable approach to the care of individuals with disabilities. By providing a detailed map of the literature and identifying critical areas for intervention, this review has the potential to directly influence the training of the next generation of physicians, preparing them to offer person-centered health care. This initiative strengthens the evidence base for curricular and pedagogical decisions and catalyzes the necessary transformation to achieve more inclusive and compassionate health care.

The next steps include conducting searches and selecting studies, extracting and synthesizing data, consulting with experts, and disseminating the results in journals and conferences. This review is expected to be completed within 12 months.

## Supplementary material

10.2196/80280 Multimedia Appendix 1 Data extraction tool.

10.2196/80280 Checklist 1 PRISMA-ScR (Preferred Reporting Items for Systematic reviews and Meta-Analyses extension for Scoping Reviews) checklist.

## Abbreviations

DS: Down syndrome
IDD: intellectual or developmental disability
JBI: Joanna Briggs Institute
MeSH: Medical Subject Headings
OSF: Open Science Framework
PRISMA: Preferred Reporting Items for Systematic Reviews and Meta-Analyses
PRISMA-ScR: Preferred Reporting Items for Systematic Reviews and Meta-Analyses extension for Scoping Reviews
