# Supplementary material for: Strengthening Undergraduate Medical Education for Inclusive Health Care for People With Down Syndrome and Intellectual and Developmental Disabilities in Medical Schools: Protocol for a Scoping Review
Source: JMIR Res Protoc. 2026 Mar 16;15:e80280. doi: 10.2196/80280 (PMC12991187; doi:10.2196/80280)
Supplement: Multimedia Appendix 1 [file resprot-v15-e80280-s001.docx]

**APPENDIX A. DATA EXTRACTION TOOL**

**Table 1. Data Extraction Form for Scoping Review**

| Section | Category | Subcategory | Details to be Extracted |
| --- | --- | --- | --- |
| 1. Study Identification | **Full Citation** | Authors | List all authors' last names and initials |
|  |  | Publication Details | Year, Journal/Source, Volume, Issue, Pages, DOI |
|  | **Publication Type** |  | Peer-reviewed journal article, thesis, dissertation, conference proceeding, government report, book chapter, etc. |
|  | **Country of Study** |  | Country where the study was conducted (specifically noting if Brazil) |
|  | **Language(s) of Publication** |  | English, Portuguese, Spanish, or other |
|  | **Funding & Conflicts of Interest** |  | Stated funding source (if any), explicit declaration of conflicts of interest (if any) |
| 2. Study Aims & Design | **Research Question(s)/Objective(s)** |  | Explicitly stated research question(s) or objectives of the included study, particularly those related to medical education for DS/IDD care. |
|  | **Study Design** |  | Quantitative (e.g., cross-sectional, quasi-experimental), Qualitative (e.g., phenomenology, grounded theory), Mixed Methods, Program Description, Experience Report, etc. |
|  | **Study Setting(s)** |  | Specific university name(s), number of institutions, type of medical school (public/private), hospital, community clinic, online learning environment, etc. (note if specific to Brazil) |
|  | **Sampling & Participants** | Sample Size (N) | Total number of participants |
|  |  | Participant Type | Medical students (specify year/phase: pre-clinical, clinical, final year), medical residents (specify specialty), recently graduated physicians (specify years post-graduation). |
|  |  | Participant Demographics | Age (mean/SD, range), Gender (% male/female), other relevant characteristics (e.g., previous experience with disability, if reported). |
|  | **Data Collection Method(s)** |  | Surveys/Questionnaires, Interviews (individual/semi-structured), Focus Groups, Direct Observation, Pre/Post-tests, Performance Assessments (e.g., OSCE), Document Analysis (e.g., curriculum documents). |
|  | **Data Analysis Method(s)** |  | Statistical analysis (specify tests), thematic analysis, content analysis, discourse analysis, mixed methods integration strategy. |
| 3. Educational Intervention/Content | **Name/Description of Program** |  | Specific course, module, rotation, workshop, seminar, interprofessional initiative, curriculum component. |
|  | **Target Audience (within medical education)** |  | Specific group receiving the education (e.g., 2nd-year medical students, Family Medicine residents). |
|  | **Duration & Frequency** |  | Total hours, number of sessions, length of rotation/module (e.g., 2-hour lecture, 4-week clinical rotation, longitudinal program over 2 years). |
|  | **Pedagogical Strategies Used** |  | Lectures, case-based learning, problem-based learning, flipped classroom, simulation (type), direct patient contact (specify type: clinical placement, home visits), community engagement, role-playing, small group discussions, reflective practice, use of technology. |
|  | **Content Focus related to DS/IDD Care** | Clinical/Biomedical Aspects | Genetics, diagnosis, common medical conditions, developmental milestones, physical exam adaptations. |
|  |  | Psychosocial/Functional Aspects | Communication strategies (e.g., with non-verbal patients), daily living skills, social inclusion, family support, patient advocacy, quality of life. |
|  |  | Ethical/Legal Aspects | Patient autonomy, informed consent, shared decision-making, disability rights legislation, addressing biases/stigma. |
|  |  | Interprofessional/Community Care | Collaboration with other professionals (e.g., therapists, social workers), referral pathways, community resources. |
|  |  | Critical Approach to Disability | Discussion of social vs. medical model of disability, challenging stereotypes. |
| 4. Outcomes Measured (related to DS/IDD care) | **Knowledge** |  | How knowledge about DS/IDD was assessed (e.g., multiple-choice questions, essays), and key findings (e.g., mean scores, proportion of correct answers). |
|  | **Attitudes/Perceptions** |  | How attitudes/perceptions toward people with DS/IDD were assessed (e.g., standardized scales, qualitative themes), and key findings (e.g., changes in stigma, empathy, comfort level). |
|  | **Skills/Competencies** |  | How skills related to DS/IDD care were assessed (e.g., clinical exam, communication, care planning), and key findings (e.g., observed performance, self-reported skills). |
|  | **Self-Efficacy/Confidence** |  | How self-efficacy/confidence in caring for DS/IDD was assessed (e.g., Likert scales), and key findings (e.g., levels of perceived competence). |
|  | **Instruments Used for Outcomes** |  | Name of specific scales, questionnaires, checklists, or other assessment tools used for each outcome type. |
| 5. Key Findings & Discussion | **Main Results Summary** | Quantitative Findings | Summarize key statistical results (e.g., significant improvements, correlations, proportions). |
|  |  | Qualitative Findings | Summarize main themes, categories, or insights derived from qualitative data. |
|  | **Effectiveness/Impact of Education** |  | Did the educational intervention achieve its stated goals? What was its impact on outcomes (knowledge, attitudes, skills)? |
|  | **Barriers & Facilitators** |  | What challenges were reported in delivering/receiving the education? What factors contributed to its success? (e.g., time constraints, faculty expertise, student engagement). |
|  | **Context-Specific Findings (Brazil)** |  | Any findings particularly relevant or unique to the Brazilian medical education or healthcare context. |
| 6. Gaps & Opportunities Identified by Authors | **Identified Gaps in Training/Research** |  | What did the authors of the included study identify as limitations in current medical training regarding DS/IDD care, or gaps in existing research? |
|  | **Recommendations for Improvement** |  | What specific recommendations did the authors make for enhancing curricula, pedagogical approaches, or clinical practice in this area? |
|  | **Suggestions for Future Research** |  | What areas did the authors suggest for further investigation? |
| 7. Reviewer's Notes | **Study Limitations** |  | List limitations of the included study as reported by its authors (e.g., small sample size, single institution, self-report bias). |
|  | **Additional Information** |  | Any other relevant details not captured above, specific nuances, data presentation format notes. |
